# Formation of Toxic Oligomeric α-Synuclein Species in Living Cells

**DOI:** 10.1371/journal.pone.0001867

**Published:** 2008-04-02

**Authors:** Tiago Fleming Outeiro, Preeti Putcha, Julie E. Tetzlaff, Robert Spoelgen, Mirjam Koker, Filipe Carvalho, Bradley T. Hyman, Pamela J. McLean

**Affiliations:** 1 Alzheimer's Research Unit, MassGeneral Institute for Neurodegenerative Disease, MGH Harvard Medical School, Charlestown, Massachusetts, United States of America; 2 Instituto de Medicina Molecular, Cell and Molecular Neuroscience Unit, Instituto de Fisiologia, Faculdade de Medicina da Universidade de Lisboa, Lisboa, Portugal; Massachusetts Institute of Technology, United States of America

## Abstract

**Background:**

Misfolding, oligomerization, and fibrillization of α-synuclein are thought to be central events in the onset and progression of Parkinson's disease (PD) and related disorders. Although fibrillar α-synuclein is a major component of Lewy bodies (LBs), recent data implicate prefibrillar, oligomeric intermediates as the toxic species. However, to date, oligomeric species have not been identified in living cells.

**Methodology/Principal Findings:**

Here we used bimolecular fluorescence complementation (BiFC) to directly visualize α-synuclein oligomerization in living cells, allowing us to study the initial events leading to α-synuclein oligomerization, the precursor to aggregate formation. This novel assay provides us with a tool with which to investigate how manipulations affecting α-synuclein aggregation affect the process over time. Stabilization of α-synuclein oligomers via BiFC results in increased cytotoxicity, which can be rescued by Hsp70 in a process that reduces the formation of α-synuclein oligomers. Introduction of PD-associated mutations in α-synuclein did not affect oligomer formation but the biochemical properties of the mutant α-synuclein oligomers differ from those of wild type α-synuclein.

**Conclusions/Significance:**

This novel application of the BiFC assay to the study of the molecular basis of neurodegenerative disorders enabled the direct visualization of α-synuclein oligomeric species in living cells and its modulation by Hsp70, constituting a novel important tool in the search for therapeutics for synucleinopathies.

## Introduction

The deposition of cytoplasmic protein inclusions is a common pathological feature of several neurodegenerative disorders. In Parkinson's disease (PD) and dementia with Lewy bodies (DLB), the main component of the inclusions is α-synuclein (aSyn), a small neuronal protein of unknown structure and function [1]. Mutations in the gene encoding aSyn have also been linked to familial cases of PD, suggesting a central role for this protein in the etiology of both sporadic and familial cases of PD [2]–[5]. In vitro, aSyn aggregation appears to be a nucleation-dependent process where a variety of intermediate species, ranging from monomers to fibrils, are formed [6], [7]. However, the mechanisms through which mutations lead to disease are not known. Multiplications of the WT aSyn gene are also linked to PD, suggesting that a simple increase in the levels of expression of the protein might be sufficient to cause neurodegeneration [8]. Several in vitro studies recapitulate this phenomenon, leading to aSyn deposition and cytotoxicity [9]–[11].

The exact link between aSyn inclusions and cytotoxicity, leading to disease onset and progression, is unclear but accumulating evidence suggests nonfibrillar dimers and oligomers of aSyn, intermediates for the formation of fibrillar inclusions and Lewy bodies (LB), play an important role in neurodegeneration [12]–[15]. Therefore, cellular processes that lead to either increased formation of dimers and/or oligomers or, alternatively, decreased clearance of these species, may be associated with aSyn-mediated toxicity.

The direct visualization of protein complexes in living cells is a challenging task, limited by the size of the complexes and the resolution of the microscopes. While macroscopic inclusions, including those containing aSyn, have been reported in several models [16], [17], the observation of smaller oligomeric and prefibrillar species has only been done indirectly, through the use of conformation-specific antibodies or biophysical techniques such as atomic force microscopy (AFM) [18]–[20]. Moreover, the effects of oligomeric and prefibrillar species on cells have not been addressed directly, and the question remains as to what the nature of the toxic species is.

In this study, we adapted a protein-fragment complementation assay (PCA) approach to observe the initial aSyn-aSyn interactions in living cells utilizing nonfluorescent fragments of GFP that can reconstitute the fluorophore when brought together by interactions between proteins covalently linked to each fragment [21], [22]. This bimolecular fluorescence complementation (BiFC) assay allows direct visualization of protein interactions in their normal cellular environment and the determination of their subcellular localization. Here, BiFC analysis allows the specific detection of a subpopulation of aSyn that forms different types of oligomeric species. Moreover, because complementation stabilizes the complex, this approach allows one to selectively enrich oligomeric species.

## Materials and Methods

### Construct Generation

aSyn BiFC constructs were generated by PCR using primers 5′GGGCTTAAGGATGTATTCATGAAAGGAC3*′*+5′GGGCTCGAGTTAGGCTTCAGGTTCGTAGTC3′ for aSyn, 5′GGGGCTAGCGCCACCATGGTGAGCAAGGGCGAGG3′ and 5′GGGCTTAAGCTGCTTGTCGGCCATGATATAG3′ for GN, and 5′GGGCTTAAGGCCACCATGGATGTATTCATGAAAGGAC3′+5′GGGCTCGAGGGCTTCAGGTTCGTAGTC3′ and 5′GGGCTCGAGAAGAACGGCATCAAGGTGAAC3′+5′GGTCTAGATTACTTGTACAGCTCGTCC3′ for GC. PCR fragments were digested, cloned into pcDNA3.1, and verified by DNA sequencing.

aSyn BiFC constructs were subcloned from pcDNA3.1 into pCMV-3Tag-8 (Invitrogen, Carlsbad, CA,USA). GN-link-aSyn was subcloned as a *SacI*/*XhoI* fragment and aSyn-GC as a PmeI/EcoRV fragment.

To make aSyn-hGLuc constructs, aSyn was subloned into the NotI/ClaI sites of constructs (kindly provided by Dr. Steven Michnick of the University of Montreal) containing optimized fragments of hGLuc (1–93; 94–185) to generate syn-hGLuc(1) and syn-hGLuc(2) fusion constructs.

### Cell Culture and Transfections

Unless otherwise stated, human H4 neuroglioma cells were maintained in OPTI-MEM medium supplemented with 10% fetal bovine serum (both from Invitrogen) and incubated at 37°C. Cells were plated 24 hours prior to transfection, growing to 80–90% confluency prior to transfection. Transfection was performed using Superfect (Qiagen, Chatsworth, CA, USA) according to the manufacturer's instructions.

For HEK (Human Embryonic Kidney) cells we used DMEM (Invitrogen) supplemented with 10% FBS and for CHO (Chinese Hamster Ovary) cells we used OPTI-MEM+10% FBS

### BiFC-GFP Reconstitution Assay

For optimal fluorophore reconstitution, transiently transfected cells were incubated overnight at 30°C [21], [22] after an initial ∼4 hour incubation at 37°C. 24 hours after transfection cells were either observed using a Zeiss LSM510 confocal microscope or harvested for preparing cell lysates.

Stable cells were also incubated overnight at 30°C prior to harvesting or microscopy.

### Gaussia luciferase protein-fragment complementation assay

To establish the syn-hGLuc PCA, we subcloned αsyn into the NotI/ClaI sites of constructs containing optimized fragments of hGLuc (1–93; 94–185) (kindly provided by Dr. Steven Michnick of the University of Montreal). Subcloning generated syn-hGLuc(1) and syn-hGLuc(2) fusion constructs which were transfected into H4 cells in a 96-well plate format as described above. Twenty-four hours after transfection, culture media was removed and replaced with phenol-red free media. Luciferase activity from protein complementation was measured in an automated plate reader at 480 nm following the injection of the cell permeable substrate, coelenterazine (20 µM) (Prolume Ltd, Pinetop, AZ) and a signal integration time of 2 seconds.

### Live cell imaging

Cells were plated on poly-D-lysine coated coverslipped 35 mm dishes (MatTek Cultureware, Ashland, MA, USA) and transfected the following day. Cells were imaged 24 hours post-transfection on a Zeiss LSM 510 confocal microscope system. Cells were observed with a 25× objective for distribution and quantification analysis and with a 63× objective for subcellular localization studies.

### Quantification of Pixel Intensities

H4 cells were plated and imaged using *Live cell imaging* methods discussed earlier. At the 24 hour timepoint cells were subjected to confocal observation using a 488 nm Laser line and a 500–550 nm emission band pass. Random fields consisting of 5–8 cells per image were collected for each dish/condition at a 25×/2× zoom setting. Prior to image collection, image and laser settings that could affect image intensity and brightness were standardized to a control or baseline dish. Adobe Photoshop was used to convert the average GFP fluorescence of each cell to average pixel intensity. Values were then averaged for each condition, and statistical differences between an empty vector/baseline condition and an experimental condition were calculated using a Student T-test.

### Native and denatured PAGE

At 24 hours post-transfection cells were washed with room temperature PBS and then harvested.

Samples to be run under denaturing conditions were lysed with lysis buffer containing Triton X-100 (0.1% Triton X-100, .15 M NaCl, 50 mM Tris pH 7.5, protease inhibitor cocktail tablet 1 tablet/10 mL (Roche Diagnostics)), sheared by passing through a 27-gauge 1 mL syringe 4–6 times, and centrifuged for 1 min at 13,000× g.

For native gels, samples were lysed with detergent-free lysis buffer (50 mM Tris/HCl pH 7.4, 175 mM NaCl, 5 mM EDTA pH 8.0 and a protease inhibitor cocktail tablet (Roche)).

Protein concentration was determined using the BCA protein assay and 20 µg of each lysate was loaded on the gel (Tris-glycine gels, Invitrogen). For denaturing conditions, SDS-PAGE was performed using Tris-Glycine SDS running buffer and SDS-sample buffer (2×, mixed with beta-mercaptoethanol at 1∶50). Native-PAGE was run with detergent-free Tris-Glycine running buffer and 2× native sample buffer (Invitrogen). Proteins were transferred to PVDF membrane (PerkinElmer) and processed for immunoblotting. Membranes were blocked in either 5% milk in TBS-T or Li-Cor blocking buffer (LI-COR,Lincoln, NE, USA) for 1 hour at room temperature. Membranes were then incubated with primary antibodies (mouse anti-alpha-synuclein, 1∶1000, BD Transduction; Rabbit anti-GFP: polyclonal, 1∶3000, Abcam) for 2–3 hours at room temperature or overnight at 4 C. After three 5–10 min TBS-T washes, membranes were incubated at room temperature for 1 hour with either IR-labeled secondary antibodies (IR800 goat anti-mouse or anti-rabbit, 1∶2000, Rockland Immunochemicals, PA,USA), Alexa 680/700 goat anti-mouse and goat anti-rabbit 1∶2000,Molecular Probes, Eugene, OR,USA) or HRP-conjugated secondary antibodies (1∶2000). After three 5–10 min TBS-T washes, immunoblots were analyzed using either the Odyssey Infrared imaging system (Li-Cor, Lincoln, NE,USA) or the ECL chemiluminescent detection system (Amhersham/GE HealthCare, Buckinghamshire, UK).

### Hoescht nuclear staining

Hoescht 33342 dye (10 mg/mL in solution, Molecular Probes/Invitrogen) in OPTI-MEM+10%FBS was applied to cells at 1 µg/mL and incubated for 30 minutes at room temperature. The media was then removed and cells were imaged in PBS.

### Cell culture, transfections, and immunocytochemistry

Human H4 neuroglioma cells (HTB-148 - ATCC, Manassas, VA, USA) were maintained in OPTI-MEM (Life Technologies, Grand Island, NY, USA) supplemented with 10% fetal bovine serum. H4 cells were passaged 24 h prior to transfection and plated in 24-well plates. Cells were transfected with equimolar ratios of plasmids using Superfect (Qiagen, Chatsworth, CA, USA) according to the manufacturer's instructions. After 24 hours, cells were washed with PBS and fixed with 4% paraformaldehyde for 10 min at RT. After washing with PBS cells were permeabilized in TBS containing 0.1% Triton X-100 for 20 min at RT. After blocking in 1.5% normal goat serum containing TBS for 1 hour, cells were incubated with primary antibody for 2 hours at RT or overnight at 4°C (anti-aSyn – syn1, BD, or anti-GFP – Abcam) followed by washing with PBS and secondary antibody incubation for 1 hour (goat anti-mouse IgG-Alexa488, 1∶300, Molecular Probes, Eugene, OR, USA; goat anti-rabbit IgG-Cy3 1∶500, Rockland Immunochemicals, Gilbertsville, PA, USA). After a final wash, slides were mounted with aqueous mounting solution (GVA, Zymed, San Francisco, CA, USA) and subjected to fluorescence microscopy.

### aSyn toxicity assay

Toxicity was analyzed 24 hr after transfection by measuring the release of adenylate kinase from damaged cells into the culture medium using the ToxiLight™ kit (Cambrex, Walkersville, MD) according to the manufaturer's protocol, using a luminescent readout.

### Fluorescent-activated cell sorting (FACS)

0.5–2.5×10 cells per dish were plated and transfected in 100 mM dishes. 24 hours post transfection Trypsin was added to each plate and neutralized with media (Opti-Mem®+10% Fetal Bovine Serum). The cell suspension was centrifuged, the supernatant aspirated and the pellet reconstituted in phosphate buffered saline (PBS). The resulting supernatant was filtered with cell strainer caps into polypropylene tubes (both from BD Biosciences). Fluorescence was measured on a FACSCanto (BD Biosciences).

## Results

**Figure 1:**
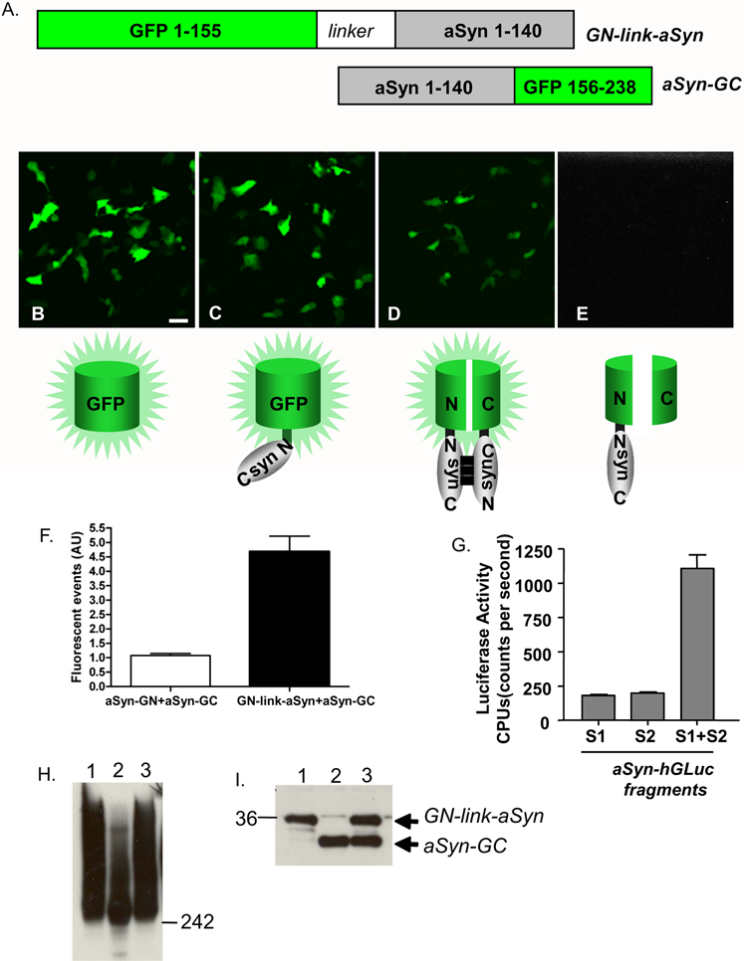
aSyn forms dimers/oligomers in cells. A. Schematic of the constructs selected for the remainder of the study. B.–E. Confocal microscopy showing the specificity of the complementation reaction driven by aSyn-aSyn interactions (Scale bar, 50 µm). F. FACS analysis of parallel vs anti-parallel aSyn-aSyn interaction. G. aSyn-aSyn interactions were also monitored using a bioluminescent-protein PCA whereby non-bioluminescent halves of G. princeps luciferase were fused to aSyn molecules. Cotransfection (S1+S2) results in a more than 5-fold increase in luciferase activity compared to background single transfections (S1 or S2), indicative of at least dimer formation. H. Immunoblot of native PAGE of cells transfected with GN-link-aSyn (1), aSyn-GC (2), and GN-link-aSyn+aSynGC (3) showing smears which are indicative of a wide range of oligomeric species. I. Immunoblot of SDS-PAGE of the same cells as in G showing the levels of expression of GN-link-aSyn and aSynGC. Immunoblots were probed with an antibody against EGFP (Abcam, Cambridge, USA).

To investigate whether aSyn forms dimers and/or oligomers in living cells we adapted a fluorescent protein-fragment complementation assay (PCA) whereby we generated a variety of fusion constructs containing non-fluorescent GFP fragments with or without a poly-linker sequence (Table 1 and Fig. 1A). Protein complementation only occurred when aSyn was fused to both fragments of GFP, and was not observed when a GFP fragment alone was expressed with GN-link-aSyn, indicating no spontaneous interaction occurred between the two GFP fragments (Table 1 and Fig. 1B–E). Complementation was stronger in cells expressing GN-link-aSyn and aSyn-GC than in cells expressing aSyn-GN and aSyn-GC, supporting an antiparallel interaction between two aSyn molecules (Table 1 and Fig. 1A–F). GN-link-aSyn and aSyn-GC constructs were used for all subsequent experiments. To ensure aSyn oligomerization was not driven by the GFP moieties, we explored a different PCA based on bioluminescence. This assay works under the same principle as the fluorescent-protein PCA except that it utilizes non-bioluminescent amino-terminal and carboxy-terminal fragments of *Gaussia princeps* luciferase (hGLuc) that can reconstitute when brought together by protein interactions [23]. Bioluminescent-protein PCA also allows direct monitoring of protein interactions in their normal cellular environment. aSyn was subloned into constructs containing optimized fragments of hGLuc (1–93; 94–185) to generate syn-hGLuc(1) and syn-hGLuc(2) fusion constructs. Transient transfection of syn-hGLuc(1) and syn-hGLuc(2) resulted in luciferase activity more than 5-fold above background (Fig. 1G). These data are consistent with the GFP-based PCA and support the formation of at least aSyn dimers.

**Table 1:** Constructs used in this study and complementation results.

N-terminal fragment	C-terminal fragment	Fluorescence signal
GN	GC	-
GN-link-aSyn	aSyn-link-GC	-
GNaSyn	aSyn-GC	-
GN-link-aSyn	aSyn-GC	+++
GNaSyn	aSyn-link-GC	-
GN-link-aSyn	GC	-
GN	aSyn-GC	-
aSyn-GN	aSyn-GC	+

Complementation occurred only with the combinations shown in green. Co-transfection of GN-link-aSyn with aSyn-GC resulted in the strongest fluorescent signal (+++) indicating the most favorable interaction. All negative controls and other combinations tested showed detectable fluorescent signal.

Under the experimental conditions used no amyloid-like, macroscopic intracellular aSyn inclusions were observed (Fig. 1 and immunucytochemistry data, not shown), suggesting that the bimolecular interaction reflected by the successful GFP complementation represents pre-aggregate intermediate species as previously suggested by FRET studies [24]; however, the fact that complementation occurs with both N/C- and C/C-terminal tags suggests that the assay can capture alternative oligomeric species.

In principle, it would be possible that both aSyn dimers and higher order oligomeric species would be detected using the BiFC assay. To study the nature of the aSyn species visualized by the BiFC assay, we employed non-denaturing (native) polyacrylamide gel electrophoresis (PAGE). Immunoblotting with both aSyn and GFP antibodies revealed a wide range of aSyn species in living cells, from dimers to high molecular weight (MW) oligomeric species (Fig. 1H). As expected, cells expressing either GN-link-aSyn or aSyn-GC (or wild-type aSyn) also contained high MW species under native conditions, however these oligomers were not fluorescent and were unable to be observed using fluorescence microscopy. Under denaturing conditions, discrete bands corresponding to monomeric GN-link-aSyn and aSyn-GC were observed (Fig. 1I).

**Figure 2:**
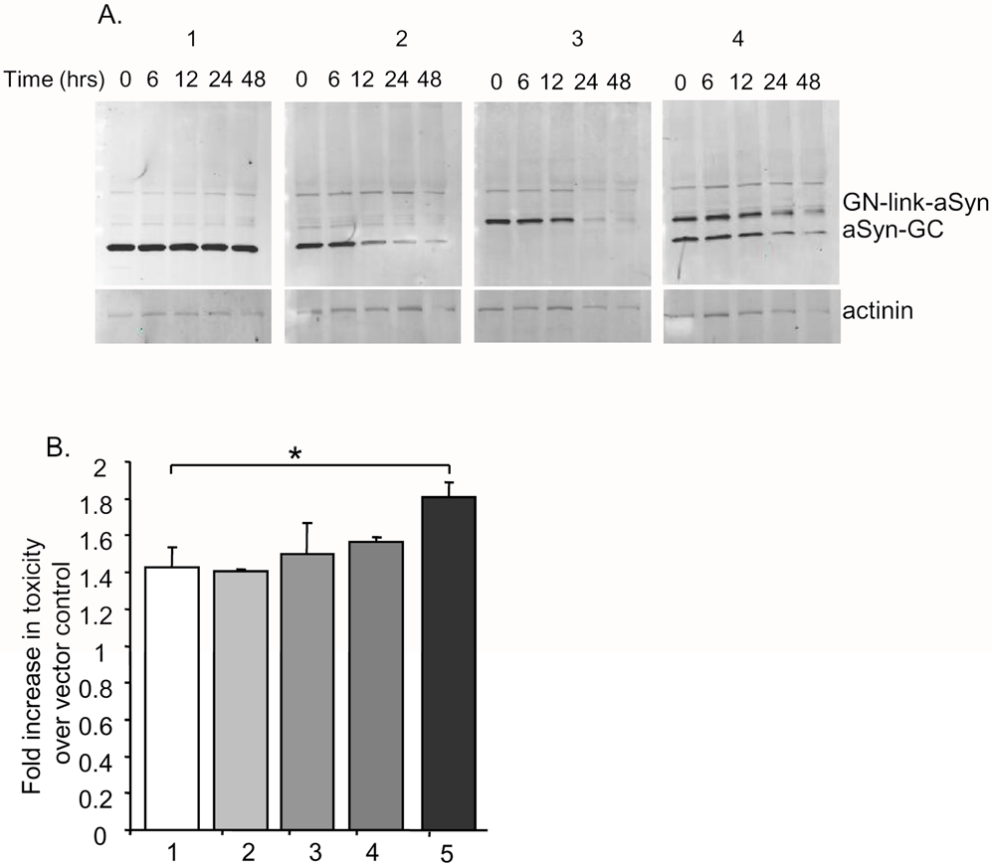
aSyn oligomers are stabilized by complementation and show increased cytotoxicity. A. The half lives of EGFP (1), aSyn-GC (2), GN-link-aSyn (3), and GN-link-aSyn+aSynGC (4) were monitored via immunoblotting of H4 cells transfected with the different constructs and treated with cycloheximide for the indicated period of time prior to cell harvesting. Immunoblots were probed with an antibody against EGFP (Abcam, Cambridge, USA).B. Toxicity assay of cells transfected with EGFP (1), WT aSyn (2), GN-link-aSyn (3), aSyn-GC (4), and GN-link-aSyn+aSynGC (5) showing that stabilization of aSyn oligomers leads to increased toxicity (*t-test, n = 3, p<0.005).

It is known that GFP reconstitution via BiFC stabilizes the protein-protein interactions of the proteins involved [22] which, in the case of aSyn, is useful because it traps the oligomeric species in a specific conformation. To assess the effect of complementation on the stability of each aSyn fusion, we performed a cycloheximide chase followed by SDS-PAGE. We found that oligomerization of aSyn increased the half life of each of the individual fusions by ∼40%. At 24 and 48 hours, the levels of GN-link-aSyn and aSyn-GC are higher when the proteins are co-expressed (i.e. when the oligomeric forms are stabilized by complementation) than when they are expressed individually (Fig. 2A).

Given that the BIFC assay traps aSyn oligomeric species in a specific conformation we next asked whether stabilized aSyn oligomeric species increased overall aSyn cytotoxicity in H4 cells. We found that, indeed, the levels of toxicity were increased in cells expressing both GN-link-aSyn and aSyn-GC when compared to cells expressing untagged aSyn or either of the GN-link-aSyn or aSyn-GC constructs alone (Fig. 2B).

**Figure 3:**
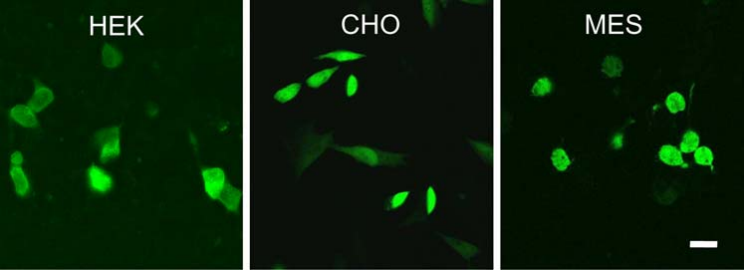
aSyn forms oligomers in cell lines of different origin. GN-link-aSyn and aSyn-GC were co-transfected into HEK, CHO, or MES23.5 cells showing complementation can occur in the different cellular environments provided by each cell line (Scale bar, 50 µm).

To determine whether different cellular environments, provided by different cell lines, could support aSyn oligomerization, we co-transfected neuronal and non-neuronal cell lines with the GN-link-aSyn and aSyn-GC constructs and studied oligomer formation using confocal microscopy. Interestingly, we found that aSyn formed oligomers in all cell lines tested (Fig. 3), suggesting the formation of oligomeric species is not the determining factor per se for the specific vulnerability of certain cell types.

**Figure 4:**
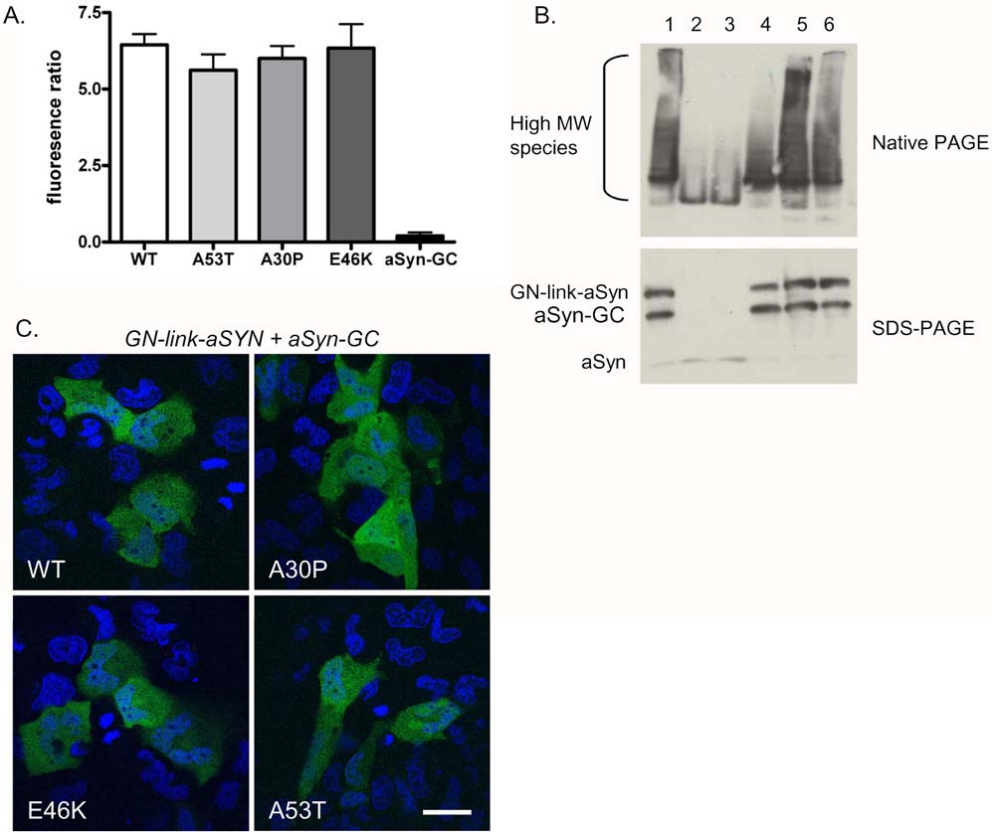
PD-associated aSyn mutations form oligomeric species. A. H4 cells were transfected with WT or mutant combinations of GN-link-aSyn+aSynGC and analyzed via flow cytometry to quantify the fluorescence intensity. The fluorescence signal was identical for WT and mutant aSyn. B. Immunoblots of native-PAGE showing oligomeric species formed by GN-link-aSyn+aSynGC (lane 1), WT aSyn (lanes 2 and 3), A53T (lane 4), A30P (lane 5), and E46K (lane 6) and the corresponding expression levels (SDS-PAGE). C. Subcellular distribution, analyzed by confocal microscopy, of WT and mutant aSyn oligomers in H4 cells showing cytoplasmic and nuclear localization (green). Hoescht staining highlight the nuclei in blue (Scale bar, 50 µm).

To assess the effect of familial PD mutations on aSyn oligomerization, we generated GN-link-aSyn and aSyn-GC constructs carrying the A53T, A30P and E46K mutations. Using the BiFC assay we found that all three mutants formed dimers/oligomers in H4 cells as assessed by GFP fluorophore reconstitution (Fig. 4A). While we were not able to detect significant differences in the oligomerization pattern via fluorescent signal, we identified different patterns in the biochemical nature of the oligomers. The A30P mutant formed high MW species to a greater degree than WT, A53T, or E46K (Fig. 4B). No statistically significant differences in cytotoxicity were observed. We then asked whether the oligomeric species formed by the WT aSyn and each aSyn mutant were differentially distributed throughout the cell. Using confocal microscopy we found all aSyn variants displayed similar subcellular distributions, with oligomers being found both in the cytosol and in the nucleus (Fig. 4C).

**Figure 5:**
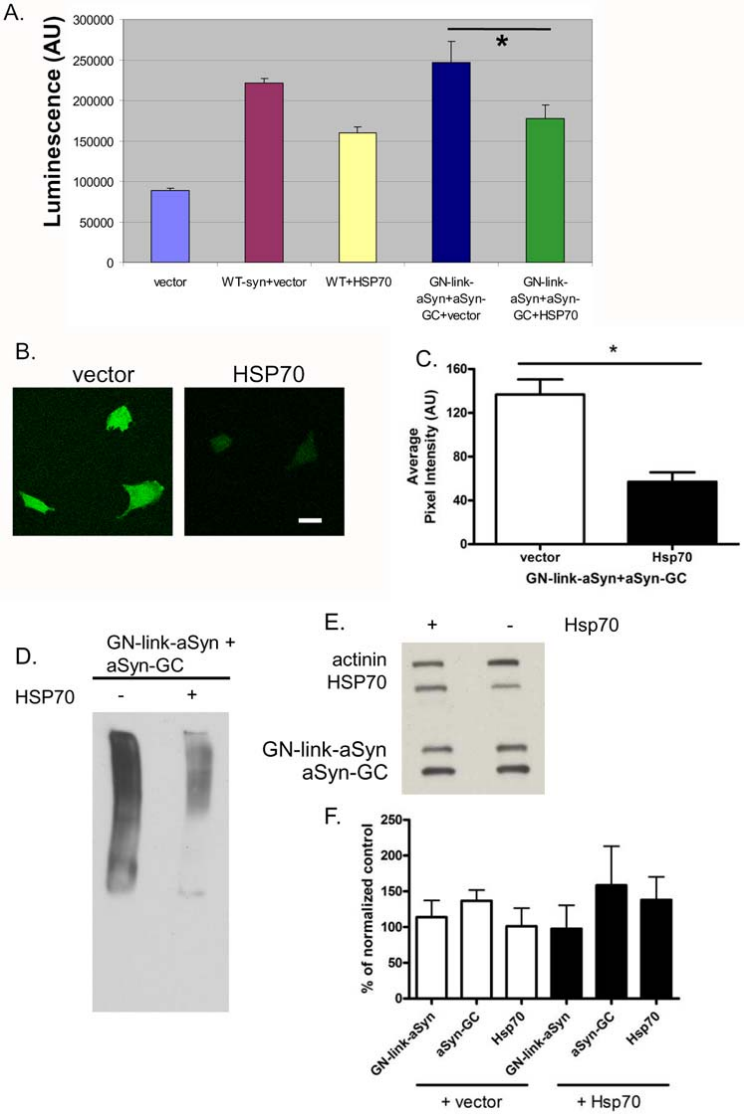
Hsp70 reduces aSyn oligomerization and toxicity in living cells. A. H4 cells were co-transfected with WT aSyn or GN-link-aSyn+aSynGC and either with an empty vector or with Hsp70. Cytotoxicity was reduced by Hsp70 (t-test, n = 3, p<0.001). B. Confocal microscopy analysis showing that overexpression of Hsp70 reduces aSyn oligomerization (Scale bar, 50 µm). C. Quantification of the pixel intensity of the same cells as in B showing a statistically significant reduction in fluorescence in cells overexpressing Hsp70 (t-test, p<0.0001). D. Immunoblot of a native PAGE of cells co-transfected with GN-link-aSyn+aSynGC and either an empty vector of with Hsp70 showing a strong reduction in high molecular weight oligomeric species by Hsp70. E. Immunoblot of an SDS-PAGE of the same samples as in E showing Hsp70 overexpression does not lead to decreased levels of GN-link-aSyn nor aSynGC. F. Quantification of the SDS-PAGE confirms that Hsp70 does not reduce the protein levels of GN-link-aSyn nor aSyn-GC.

We and others have previously shown that overexpression of Hsp70 reduces aSyn toxicity and aggregation in H4 cells and in animal models of PD [25], [26], [#2463,27]. To investigate whether the toxicity of aSyn dimers/oligomers could be reduced by molecular chaperones, we co-transfected cells with GN-link-aSyn, aSyn-GC and either empty vector or Hsp70. Cells co-expressing Hsp70 displayed reduced cytotoxicity, demonstrating the protective effect of this protein towards the toxic effects exerted by aSyn dimers/oligomers (Fig. 5A).

We next asked whether the rescue of cytotoxicity by Hsp70 was associated with an effect on aSyn oligomerization. We found that overexpression of Hsp70 reduced aSyn oligomerization in living cells by ∼50% (Fig. 5B, C), corresponding with a clear reduction in high MW species on a native PAGE (Fig. 5D). Importantly, the reduction of aSyn oligomerization was not simply explained through increased clearance of the monomeric forms, as the levels of GN-link-aSyn and aSyn-GC were not reduced (Fig. 5E, F). Thus Hsp70 selectively leads to clearance of aSyn in an oligomeric conformation rather than a monomeric conformation.

## Discussion

Protein misfolding and deposition are associated with several brain disorders, including Alzheimer's disease (AD) and PD, the two most common neurodegenerative diseases. Fibrillar, macroscopic inclusions made up primarily of aSyn, called LBs, are found in the brains of patients with PD and other diseases collectively known as synucleinopathies. The intermediary species that precede amyloid formation are thought to be more toxic than the inclusions themselves, but studying these presumed toxic species in cells has been difficult. In vitro, purified aSyn forms oligomeric species prior to the formation of larger structures with amyloid-like properties [7], [18], [19], [28], [29], but in living cells it has not been possible to study the range of intermediary species that fall between monomeric and aggregated forms of the protein. This has obscured the understanding of the molecular mechanisms leading to neurodegeneration and the development of efficacious therapeutics directed at eliminating the source of toxicity.

Recently, novel protocols enabled the generation of aSyn oligomers from purified proteins, and their effects on cells suggested heterogeneous populations lead to cell death [30]. The present study takes advantage of a novel assay that enables the direct visualization of protein-protein interactions in the context of a living cell, using GFP as a reporter [22]. We sought to investigate, in living cells, the initial interactions that lead to the formation of intermediary aSyn oligomeric/pre-aggregated species. We monitored aSyn dimerization by fusing non-fluorescent fragments of GFP (amino acids 1–155 or 156–238) to the N- and C-termini of aSyn molecules and observed reconstitution of the functional GFP fluorophore only in conditions where the two fragments were brought together by aSyn-aSyn interactions. aSyn dimers formed throughout the cell, including in the nucleus, suggesting different subcellular environments can offer a productive environment for dimerization. Importantly, aSyn has recently been shown to occur in the nucleus and to bind directly to histones [31]. Our data are consistent with this finding and demonstrate that aSyn dimers and oligomers are also present in the nucleus.

The BiFC assay is known to lead to the stabilization of the interaction between the proteins of interest due to the reconstitution of the GFP moiety [22]. This property of the assay allowed us to address a central question in the field which is whether pre-aggregated species (dimers, trimers, oligomers, etc) are more or less toxic than WT aSyn [9], [16], [17]. Importantly, we found that stabilizing aSyn oligomeric species led to a statistically significant increase in cytotoxicity (∼20%). Using native PAGE we could detect aSyn oligomeric species for all the constructs tested, including WT aSyn, as expected, but when aSyn oligomers were stabilized by complementation the half lives of the oligomeric species were increased, along with a concomitant increase in cytotoxicity. Importantly, the converse experiment also implicates oligomeric forms of aSyn in toxicity. Overexpression of the chaperone Hsp70 selectively impacts high MW species (rather than monomeric forms of aSyn) and has a clear cytoprotective effect.

The possibility exists that the fusion of GFP fragments to aSyn may influence its behavior, however in similar experiments performed with fragments of humanized Gaussia luciferase [23] fused to aSyn, the data presented herein were successfully replicated, which suggests that GFP is not responsible for driving the observed interactions. An important aspect of the BiFC assay is that oligomeric forms of aSyn can be directly visualized in living cells, providing insight into subcellular localization. Interestingly, in this case, the aSyn oligomers are strongly visualized in the nucleus.

Overall, our data demonstrate that the formation of dimeric and oligomeric aSyn species, both of which are thought to precede the formation of larger intracellular inclusions, are central steps towards cytotoxicity which can be targeted through the activity of molecular chaperones, such as Hsp70. Further studies will be necessary to investigate the formation of aSyn dimers and oligomers in animal models and to assess their toxicity. Nevertheless, through the use of this novel powerful assay, our work opens new avenues for investigating the precise nature of the toxic aSyn species and for using oligomeric species as a target for therapeutic intervention in PD and other synucleinopathies.
