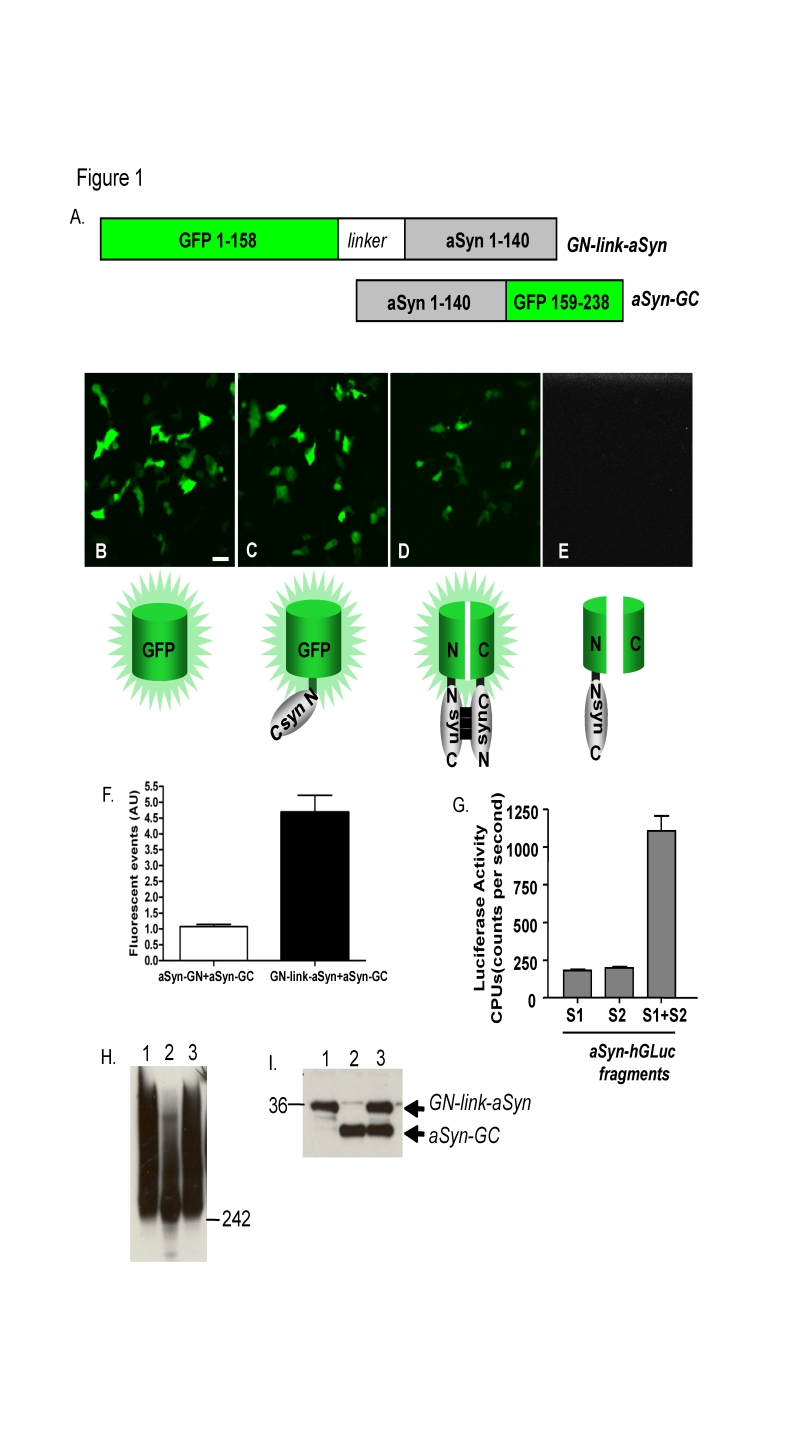# Correction: Formation of Toxic Oligomeric α-Synuclein Species in Living Cells

**DOI:** 10.1371/annotation/9282f173-df82-4b70-9120-b4e62b3dacb1

**Published:** 2008-05-16

**Authors:** Tiago Fleming Outeiro, Preeti Putcha, Julie E. Tetzlaff, Robert Spoelgen, Mirjam Koker, Filipe Carvalho, Bradley T. Hyman, Pamela J. McLean

There was an error in the numbering in Figure 1A. The corrected figure is available here:

**Figure:**